# Coal-Based Semicoke-Derived Carbon Anode Materials with Tunable Microcrystalline Structure for Fast Lithium-Ion Storage

**DOI:** 10.3390/nano12224067

**Published:** 2022-11-18

**Authors:** Yaxiong Liu, Xing Guo, Xiaodong Tian, Zhanjun Liu

**Affiliations:** 1 CAS Key Laboratory of Carbon Materials, Institute of Coal Chemistry, Chinese Academy of Sciences, Taiyuan 030001, China; 2 Center of Materials Science and Optoelectronics Engineering, University of Chinese Academy of Sciences, Beijing 100049, China

**Keywords:** semicoke, carbon anode, turbostatic phase, short-ordered defective structures, large interlayer spacing, fast lithium storage

## Abstract

Fast charging capability is highly desired for new generation lithium-ion batteries used in consumer-grade electronic devices and electric vehicles. However, currently used anodes suffer from sluggish ion kinetics due to limited interlayer distance. Herein, the coal-based semicoke was chosen as precursor to prepare cost-effective carbon anodes with high-rate performance through a facile pyrolytic strategy. The evolution of microstructure and its effect on electrochemical performance are entirely studied. The results show that large number of short-ordered defective structures are generated due to the occurrence of turbostatic-like structures when pyrolyzed at 900 °C, which are propitious to large interlayer distance and developed porous structure. High accessible surface area and large interlayer spacing with short-ordered defective domains endow the sample treated at 900 °C under argon (A900) with accelerated ion dynamics and enhanced ion adsorption dominated surface-induced capacitive processes. As a result, A900 delivers high capacity (331.1 mAh g^−1^ at 0.1 A g^−1^) and long life expectancy (94.8% after 1000 cycles at 1 A g^−1^) as well as good rate capability (153.2 mAh g^−1^ at 5 A g^−1^). This work opens a scalable avenue to fabricating cost-effective, high-rate, and long cycling life carbon anodes.

## 1. Introduction

The global environmental problems and energy crisis triggered by the overexploitation and utilization of fossil fuels have aroused great attention in developing the new energy industry. As an important energy storage technology, lithium-ion batteries (LIBs) have dominated the battery market of consumer electronics, smart grids, electric vehicles (EVs), etc., owing to their high energy density, good cycle performance, low self-discharge rate, etc. [1,2,3]. In the perspective of the burgeoning development of EVs and consumer-grade electronic devices, it is urgently desirable to develop cost-effective energy storage systems with long cycling lifespan and fast charging capability to ameliorate endurance anxiety [4]. However, the fast charging performance of state-of-the-art LIBs is afflicted by the sluggish ion kinetics caused by the limited interlayer distance of commercially used graphite anodes [5]. Generally, the fostered reaction kinetics of an anode depend on the diffusion ability of lithium ions and electrons within the electrode bulk. In this line, electrode materials with tailor-made structures which can provide abundant active sites, short transmission routes, and favorable electronic/ion conductivity are unequivocally required [6].

**Figure 1:**
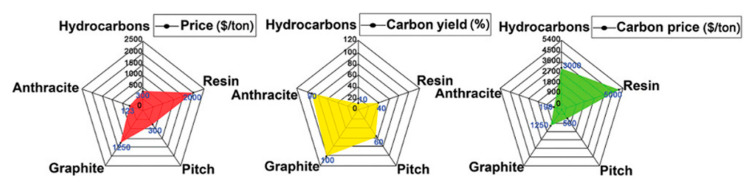
The comparison of price, carbon yield, and carbon price of selected precursors for carbon-based anodes for batteries. Reprinted with permission from Ref. [9]. Copyright 2017 John Wiley and Sons.

Recently, porous carbonaceous materials with large interlayer spacing and plenty of active sites have aroused broad attention for high-rate performance LIBs. Resins, polymers, biomass, coal, and their derivatives have been used as precursors to prepare porous carbon, and exhilarating progress has been made recently [7,8]. However, regarding electrochemical properties, such as specific capacity, cycling life and rate performance, the cost and environmental impact should also be seriously considered. Concretely, biomass carbon is limited due to its raw material transportation and source as well as low carbon yield. The main reasons for the limitation of resins and polymers are price factor and low carbon yield. Nevertheless, coal-based carbon shows great advantages in price and carbon yield, especially in carbon price convenience. Wang et al. summarized that coal-based materials are more suitable as a precursor considering price, carbon yield, and obtained carbon price (Figure 1) [9].

Due to its cost efficiency and high carbon yield, coal and its derivatives—such as coal tar pitch, semicoke, and coke—have been widely studied for energy storage [10,11,12,13,14]. For example, bituminous coal has been heated to different temperatures (800 °C, 1000 °C, and 1200 °C) under argon and nitrogen to prepare coal-based porous carbon for sodium ion and lithium ion storage. The authors found that the samples treated at low temperature display satisfactory capacity (409 mAh g^−1^) [15]. Zhong et al. transformed anthracite and semicoke into multilayer graphene spheres which had high reversible capacities of 389.8 mAh g^−1^ and 401.4 mAh g^−1^, respectively [16]. Inspired by the above encouraging progresses, Li et al. treated the low-cost coal coke with concentrated HNO_3_ followed by high-temperature calcination, and the obtained porous carbon delivers 273 mAh g^−1^ at 0.2 C with outstanding magnification performance (257 mAh g^−1^ at 10 C) [10]. This work could be regarded as an intimation that coal coke has the potential to fabricate high-rate performance carbonaceous anode material for LIBs. However, it is unfortunate that concentrated acid and high treating temperature were used, as they are environmentally unfriendly, require high energy consumption, and run counter to the environmental protection target. Thus, it is of great importance to find a low-cost, environmentally friendly, and practical way to convert coal coke into high-performance anode materials for LIBs.

Hereby, semicoke was adopted as carbon precursor due to the combination merits of intrinsic macropore structure and high fixed carbon content, which are favorable for the preparation of hierarchical porous structure with high carbon yield. In this work, low temperatures of 600 and 900 °C were used. The samples were synthesized under argon and a hydrogen/argon mixture to probe the microcrystalline evaluation. The results demonstrate that turbostatic stacking structure occurs upon the increase in heating temperature from 600 °C to 900 °C, leading to the formation of more disordered structures with short-range ordered domains. Coupling with large interlayer distance, optimized pore size distribution, and more accessible active surface, the obtained anode material treated at 900 °C under argon presents rapid ion kinetics and enhanced capacity as well as good rate capability and long life. Our work provides a simple and low-energy-consumption approach to scale up low-cost coal-based anode materials with high performance.

## 2. Materials and Methods

### 2.1. Materials Preparation

Firstly, the semicoke bulks, which were obtained from Shenmu Chuangyou Energy Technology Co., Ltd. (Yulin, China), were ground into powders and filtered with a 200-mesh net. Then, these powders were purified successively with 30% hydrochloric acid (HCl) and 10% hydrofluoric acid (HF) to remove ash. After washing, the neutralized materials were dried at 100 °C for 12 h and denoted as SC. Afterwards, the prepared SC was heated to 600 °C and 900 °C for 1 h under argon or hydrogen/argon (5% H_2_) flow, respectively, with a gradient of 5 °C min^−1^. The resulting products were named A600, A900, H600, and H900, respectively. A means argon, and H represents hydrogen/argon atmosphere.

### 2.2. Material Characterization

The topography of samples was characterized using a JMS-7001F (Jeol, Tokyo, Japan) desktop scanning electron microscope (SEM) and JEM-2001F (Jeol) transmission electron microscopy (TEM). The selected area electron diffraction (SAED) patterns were also recorded. The crystal information of the sample was collected using D8 ADVANCE X-ray diffraction (XRD) (Bruker, Bremen, Germany) and a LabRAM HR Evolution Raman spectrometer (Horiba, Kyoto, Japan). The textual structure was obtained via the Brunauer–Emmett–Teller (BET) technique and the density functional theory (DFT) method. X-ray photoelectron spectroscopy analysis (XPS) and elemental analysis (EA) were performed on an AXIS ULTRA DLD (Kratos, Manchester, UK) and a Vario EL CUBE (Elementar, Frankfurt, Germany) elemental analyzer to probe the surface’s elemental composition and content.

### 2.3. Electrochemical Characterization

The electrodes were prepared by coating copper foil with the mixture of prepared samples, polyvinylidene fluoride, and carbon nanotubes with a mass ratio of 8:1:1, followed by drying at 80 °C for 12 h under vacuum. The mass loading of active material was kept in the range of 1.05–1.20 mg cm^−2^. The electrolyte was 1.0 M LiPF_6_ in EC: DEC (1:1, vol%) with 5.0% FEC adopted as electrolyte. Thin lithium foil and a piece of Celgard 2400 membrane (Celgrad, Charlotte, NC, USA) were used as the counter-electrode and the separator, respectively. A battery measurement device (LAND, CT2001A, Landt Instruments, Wuhan, China) and a Chenhua electrochemical station (CHI660C, Chenhua, Huangdu Town Shanghai, China) were used to collect the electrochemical data. Galvanostatic charge–discharge (GCD) tests and cyclic voltammetry (CV) tests were carried out between 0.01 and 3.0 V vs. Li^+^/Li. Electrochemical impedance spectroscopy (EIS) was achieved between 10 mHz and 100 kHz, and the obtained data were fitted using Zview impedance software (Scribner Associates, Inc., Version 3.2c, Charlottesville, VA, USA).

## 3. Results and Discussion

### 3.1. Microstructure and Morphology

**Figure 2:**
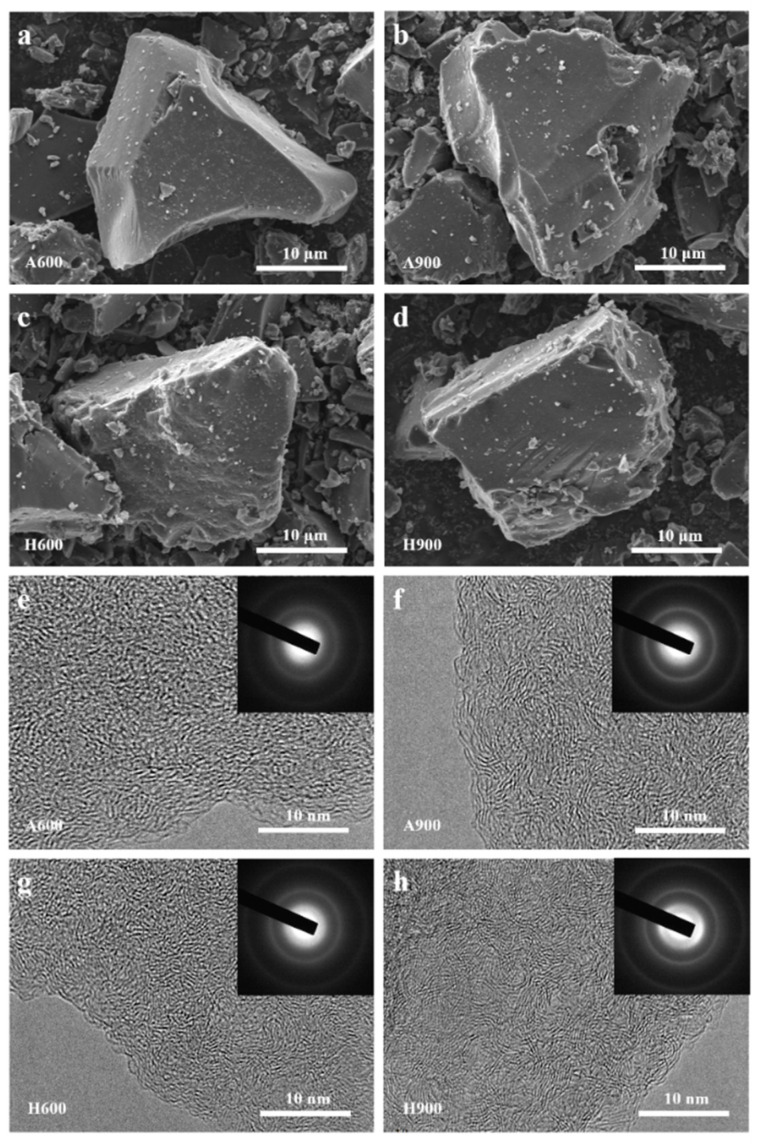
SEM images of A600 (**a**), A900 (**b**), H600 (**c**), and H900 (**d**); TEM images of A600 (**e**), A900 (**f**), H600 (**g**), and H900 (**h**).

Table S1 lists the proximate analysis of semicoke bulks. High fixed carbon content (84.53%) and low levels of ash (4.80%) pave the way for their potential application for energy storage in valuable carbon electrodes. On the basis of the results shown in Figure S1 and Table S2, one can judge that apart from C, O, and S elements, impurities such as Ca, Al, Si, and Fe elements can be endorsed. After washing with HCl and HF, these impurities can be removed. The detected Cl element should be ascribed to the residual Cl^-^ in HCl during washing. Compared to SC (Figure S2), the irregular grains were well inherited after calcination at 600 °C and 900 °C even under different atmospheres, suggesting that treatment temperature has a minimal effect on the morphology of obtained carbons (Figure 2a–d). The smoother surface of calcinated samples should be rationalized by the decomposition of volatile organic compounds in SC. The blurry cricoid SAED patterns presented in the insert of Figure 2e–h revealed the amorphous nature of prepared samples. High-resolution transmission electron microscopy (HRTEM) is a powerful tool for estimating the evolution of microcrystalline structures in samples. As illustrated in Figure 2e,g, the carbonaceous materials prepared at 600 °C (A600 and H600) display distinct amorphous structures. Upon the increase of carbonization temperature, more turbo-like short-ordered structures (graphitic domains) occur (Figure 2f,h). According to the report by Inagaki and Kang, this kind of stacking structure can be an index to turbostatic stacking architecture, which is a transitional structure between amorphous structure and graphitized structure, having an important impact on the microcrystalline size development of the materials [17]. Unlike A600 and H600, which show inconspicuous change in interlayer distance, H900 exhibited more significant layer spacing reduction compared to A900, indicating that the presence of oxygen groups and defects will inhibit the transition from the amorphous phase to the graphitic phase due to the occurrence of the turbostatic stacking phase. The work of Fromm et al. on biomass-based hard carbon also confirms that the presence of the turbine phase is not conducive to generating more ordering structure in a certain temperature range [18].

**Figure 3:**
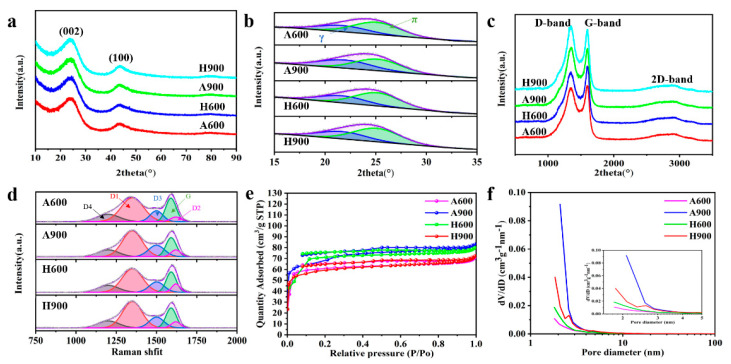
The XRD patterns (**a**) and corresponding fitted spectra (**b**), the Raman spectra (**c**) and the fitted Raman spectra (**d**), the N_2_ adsorption–desorption isotherms (**e**), and the pore size distribution (**f**) of the samples.

To further investigate the effects of temperature and atmosphere on microcrystalline evolution, especially on the turbostatic stacking structure development, XRD and Raman spectra were conducted. The XRD patterns shown in Figure 3a,b and Figure S3 divulge crystalline changes after certain treatments. All samples possess two distinguished diffraction peaks centered around 24.5° and 43°, indexing as (002) and (100) planes of carbon, respectively [19]. Unlike previously reported carbon, for which the (002) peak shifts to a higher diffraction angle with improved temperature [20,21,22], the peak of the (002) plane herein shifts to a lower angle when the carbonized temperature increases from 600 °C to 900 °C due to the existence of the turbostatic stacking phase (Table 1). The calculated interlayer spacing (d_002_) based on the Bragg equation augments this slightly regardless of if the sample was prepared under argon or mixed atmosphere [23]. The larger interlayer distance of the samples compared to commercial graphite (0.335 nm) endows these materials with fast Li^+^ diffusion, which is conducive to the improvement of rate capability. Notably, compared to the samples treated under argon, the H600 and H900 exhibit smaller d_002_ due to reduced oxygen functional groups and edge defects (Table S3) [24]. The atomic ratio calculated based on XPS results shown in Table S5 also verified this tendency.

Expect for d_002_, the average crystalline size (L_a_) and thickness of stacking (L_c_) can also be used to probe the microcrystalline structure [25]. With the increasing temperature, L_a_ and L_c_ enlarged, indicating that high temperature is inclined to accelerate the development of microcrystalline size. Owing to the fact that H_2_ will cost oxygen groups and unstable carbon at the edge of graphene layers, the samples treated by hydrogen/argon show larger L_a_ and L_c_ values, indicating enhanced ordering structure.

In order to grasp a deeper insight into microstructure evolution, the asymmetric broad peak between 15–26° was deconvoluted into π-band and γ-band, representing graphitic carbon and disordered carbon, respectively, Figure 3b [26]. The structural parameter (I_g_), standing for the amount of ideal graphite carbon, could be evaluated based on the enclosed area of the π- and γ-bands according to Equation (1) [26]:

I_g_ = A_π_/(A_π_ + A_γ_) (1)

where A_π_ and A_γ_ stand for the integral areas of the π-band and γ-band, respectively. Higher values of I_g_ for H600 and H900 in comparison to A600 and A900 suggest the enhanced ordered structure after hydrogen/argon treatment (Table 1). In addition, the R value, referring to the ratio of the height B and the baseline A of the (002) peak, is also used to evaluate the number of carbon layers arranged as single layer along the c-axis [27]. Generally, larger R means a more stack state [28,29]. As illustrated in Figure S3 and Table 1, the calculated R-values for A600, A900, H600, and H900 show the same trend as L_c_ from XRD analyses. Taking the small variations in R and the larger L_a_ of H600 and H900 compared to the samples treated under argon into consideration, it is deduced that H_2_ reduction is more efficacious in improving the molecular structure along the a-axis than along the c-axis [24].

Raman spectroscopy was further conducted to distinguish and analyze the development of the graphitic structure. As displayed in Figure 3c, two obvious peaks located at 1350 cm^−1^ (D-band, relating to the defects in the sp^2^-hybridized carbon at edges [18]) and 1590 cm^−1^ (G-band, representing the in-plane stretching vibration of C=C (νC=C) [30]) along with a small hump at around 2670–2900 cm^−1^ (2D-band) can be confirmed in all prepared samples. The existence of a 2D-band indicates the partial graphitization of the samples [31,32,33]. The weak development of the graphitic structure and domain size can be further detected from the partial overlapping of the D-band and G-band [34]. To gain more structural information hidden in the overlap, the Raman spectra in the range of 950–1750 cm^−1^ were deconvoluted into five peaks located at around 1200, 1350, 1500, 1585, and 1620 cm^−1^, representing A_1g_ breathing mode of the sp^2^-sp^3^ hybrid structure (D4 band), A_1g_ breathing mode of disordered graphite lattice (D1 band), amorphous structure or organic molecule (D3 band), E_2g_ stretching vibration mode of graphite lattice (G band), and few-layer graphene (D2 band) [35,36], respectively (Figure 3d). The following four formulas are used to analyze the Raman peak splitting data of the samples.

F_1_ = I_G_/I_all_ (2)

F_2_ = I_D1_/I_G_ (3)

F_3_ = (I_D1_ + I_D2_)/I_G_ (4)

F_4_ = (I_D1_ + I_D2_ + I_D4_)/I_G_ (5)

where F_1_–F_4_ represent the amount of graphite crystal, the carbon content at the edges of graphite crystal, the defect degree of graphite crystal, and the disordered carbon crystallite content, respectively [26]. I_x_ refers to the integral area of x peak (x = D_1_, D_2_, D_3_, and D_4_) while I_all_ stands for the sum of the integrated area of all peaks.

As presented in Table 2, the value of F_1_ decreases, while F_2_, F_3_, and F_4_ increase when the temperature elevates from 600 °C to 900 °C under same atmosphere, indicating reduced graphite crystal content and the development of disordered structure or defects in obtained carbon materials with an increase in temperature, which is well matched with the XRD results. The existence in the turbostatic stacking structure could be responsible for this. Additionally, as anticipated, the partially disordered structure and/or defects vanished after being treated by hydrogen/argon atmosphere, resulting in improved F_1_ value.

The treating temperature and atmosphere may also influence textural structure of prepared carbons. As shown in Figure 3e, all samples manifest similar N_2_ adsorption and desorption isotherms with different capacities. The isotherm curve exhibits a combination of Type I and H_4_ hysteresis loop characteristics, indicating the coexistence of microporous and mesoporous structures [37]. More precisely, the sharp uptake at the low P/P_0_ region refers to the existence of micropores. The plateau at the medium region stands for the appearance of mesopores. The uplift at the high region is characteristic of the presence of macropores, which may come from the gap between particles and/or from the matrix. The hierarchically porous structure can be further validated by the corresponding pore size distribution presented in Figure 3f. The detailed data were summarized in Table S4, from which one can find that the specific surfaces of A600, A900, H600, and H900 are 195.4, 226.3, 216.7, and 184.9 m^2^ g^−1^, respectively. A900 exhibits an intensive distribution of ultramicropore centered below 1 nm, which will boost the adsorption of Li^+^ [38]. Additionally, the most abundant mesopores with pore sizes larger than 2 nm are propitious to the superior ion diffusion capability at the deeper micropore surface of the electrode. Proper specific surface area and pore size distribution are inclined to the adsorption of Li^+^ on the electrode and the penetration of electrolyte ions.

**Figure 4:**
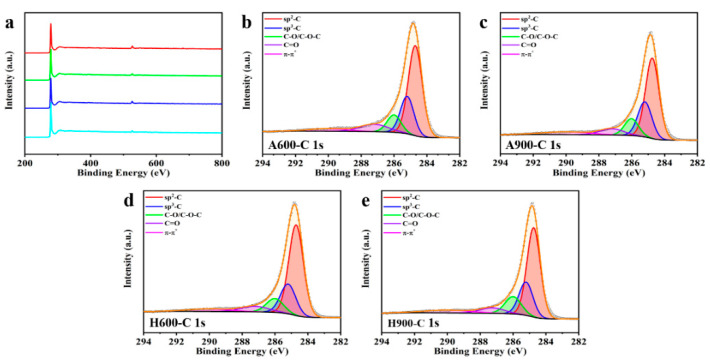
XPS overall scans (**a**) and high-resolution C 1s spectra of A600 (**b**), A900 (**c**), H600 (**d**), and H900 (**e**).

As presented in Figure 4a, all samples show obvious C 1s and O 1s signals; no S element can be detected by XPS due to its small content in the matrix. As illustrated in Table S5, the content of O reduces when the temperature increases. Hydrogen treatment can further consume oxygen functional groups, leading to lower O content in H600 and H900, which is consistent with EA analyses. To determine the chemical bond configuration of carbon and oxygen for the samples, the high-resolution C 1s peaks were fitted as sp^2^ carbon (graphitic carbon, 284.7 eV), sp^3^ hybridized carbon (defect peak, 285.4 eV), C–O/C–O–C (286.2 eV), C=O (287.2 eV), and π-π* shake-up satellites (π-π*, 290 eV) [35,39,40,41], respectively. It is universally known that the honeycomb-like graphene layer is made up of C atoms containing the sp^2^ bond, while the sp^3^-bonded carbons are usually located in defect areas. As shown in Table 3, the sp^3^-bonded carbon atoms gradually increase with the increase in calcination temperature, which represents an increase in defects, which correlates with the results of Raman and XRD. Similarly, there are fewer defects in the samples treated under hydrogen/argon atmosphere than under argon atmosphere. Meanwhile, the relative content of π-π* increases, while those of the C-O bond and C=O bond decrease, indicating a reduction in defects [39]. XPS analysis results further verified our assumption that the removal of the oxygen functional group caused more carbon defects (sp^3^ hybridized carbon) and that calcination in hydrogen atmosphere helps to reduce carbon defects.

### 3.2. Electrochemical Performances

**Figure 5:**
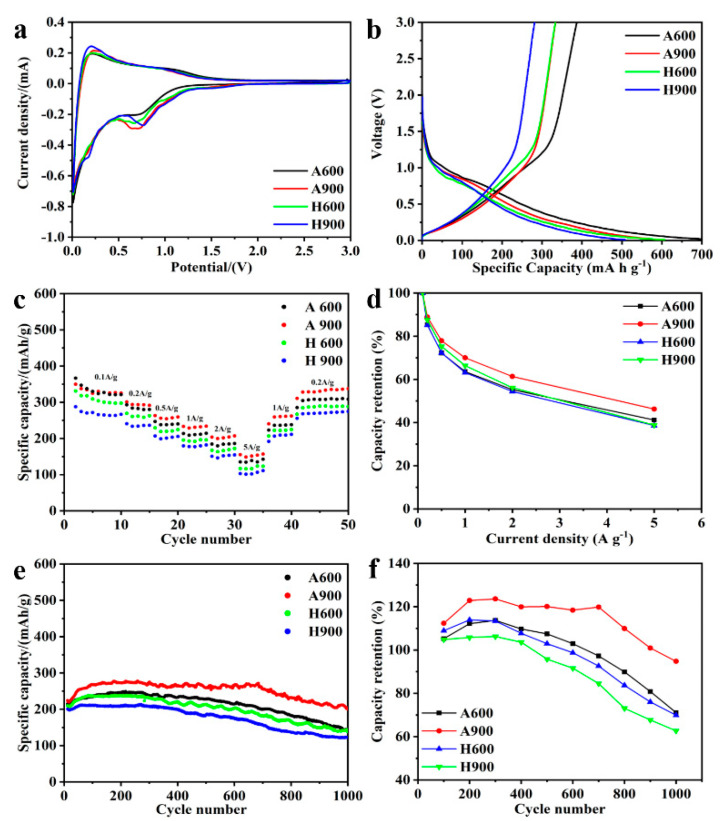
The initial CV curves of the samples at 0.2 mV s^−1^ (**a**), comparison of the initial GCD profiles of the samples at 0.1 A g^−1^ (**b**), rate performance (**c**) and normalized capacity retention (**d**) in dependence of current density, and the cycling performance (**e**) and capacity retention (**f**) from 100 to 1000 cycles cycled at 1 A g^−1^ of the samples.

Figure S4 and Figure 5a present the CV curves of obtained anodes tested at 0.2 mV s^−1^. All electrodes display typical features of the disordered hard carbon [41,42,43]. In the initial CV scan, conspicuous cathodic peaks at around 0.3 and 0.7 V can be perceived as the decomposition of electrolyte and the formation of the solid electrolyte interphase (SEI) layer [44], respectively, but these radically disappear in the following cycles. The sharp peak below 0.1 V relates to the intercalation of Li^+^ into the carbon layer. In anodic sweeping, the oxidation peak around 0.2 V is deemed to be the reversible deintercalation of Li^+^. The nearly overlapped CV curves of the following cycles verify the good reversibility of these electrodes. The broad peak observed between 0.7–1.3 V refers to the reversible reaction between oxygen groups (such as ester and carbonyl groups) and Li^+^ [45]. The A600 electrode shows the strongest peak intensity around 0.7–1.3 V, which is in line with the highest oxygen content within it.

Figure 5b presents the comparison of the initial lithiation and delithiation processes of all samples tested under 0.1 A g^−1^. By comparison, the materials obtained through calcination under argon show higher capacity owing to their high oxygen group contents [46]. The plateau of the first discharge profile can be ascribed to the formation of SEI film. The plateau region disappeared during the following cycles (Figure S5), which is well in correlation with CV curves. As displayed in Figure S5, the plots of the 5th and 10th charge/discharge plots shown overlap almost completely, indicating excellent electrochemical reversibility. The sloping voltage profiles evidenced the characteristic of disordered structure in obtained carbonaceous materials [47]. As illustrated in Table S6, the initial coulombic efficiencies of A600, A900, H600, and H900 are 52.54%, 55.66%, 54.94%, and 55.36%, respectively, with irreversible capacity losses of 349.6, 265.9, 273.1, and 226.9 mAh g^−1^, respectively. Large specific surface area and oxygen functional groups, which consume more electrolyte in the initial cycle to form stable SEI layers, should be responsible for the obvious irreversible capacity loss. The coulomb efficiency increases gradually in the following cycles. The coulombic efficiency of all samples reaches up to 92% in the second cycle. Notably, more than 98% can be achieved after 10 cycles due to the formation of stable SEI film.

Enlarged interlayer spacing, ultramicropore, carbon defects, high surface area, and the sloping characteristic of GCD plots portend fast Li^+^ diffusion. Thus, the rate capability was investigated. Figure 5c presents an overview of the specific capacity independent of the current load, ranging from 0.1 to 5.0 A g^−1^. The capacities of A600, A900, H600, and H900 at 0.1 A g^−1^ are 332.8, 331.1, 308.2, and 269.7 mAh g^−1^, respectively. A600, with lower specific surface area but high oxygen proportion, delivers comparable discharge capacity to A900, authorizing the positive role of oxygen functional groups in energy storage [45]. The specific capacity decreases gradually with the increase of current load. The specific capacities decrease to 137.1, 153.2, 119.1, and 104.7 mAh g^−1^, respectively, when the current increases up to 5 A g^−1^, with capacity retentions of 41.19%, 46.25%, 38.66%, and 38.81%, respectively. H600 and H900 show inferior retention compared to A600 and A900. The reduced carbon defects, oxygen functional groups, and interlayer spacing, which induce limited Li^+^ adsorption and diffusion, should be responsible for the capacity degradation of H900 compared to H600. Moderately ultramicropore and a more disordered structure with larger interlayer spacing endow A900 with superior capacity even at 5.0 A g^−1^ (11.7% higher than that of A600). Figure 5d lists the capacity retention as a function of 0.1 A g^−1^, from which one can find that A900 showed higher capacity retention at different current densities compared toother samples. It is noteworthy that the average discharge capacities recovered to higher levels than before when the current was set back to 0.2 A g^−1^, suggesting excellent electrochemical stability of the samples.

Cycling durability is an important index for the fabrication of high-performance LIBs. Figure 5e illustrates the cycling endurance of the four electrodes at 1 A g^−1^. The slightly increasing capacity against cycle number is evidenced for all samples during the initial 100 cycles, relating to the enhanced Li^+^ diffusion kinetics induced by high-rate lithiation-induced reactivation and the optimization of the stable SEI [48], which is a common phenomenon in porous carbon anodes with high specific surface area [49,50,51]. A900 delivers more than 100% of initial capacity even after 900 continuous cycles, while the others decay below their initial capacity quickly (Figure 5f). Notably, A900 anodes can maintain a high reversible capacity of 209.5 mAh g^−1^ with a retention of 94.8% even after 1000 cycles, validating the rapid ion kinetics and good structural stability of A900. After 1000 cycles, the average capacity decay rates per cycle of A600, A900, H600, and H900 were 0.029%, 0.005%, 0.030%, and 0.037%, respectively. It is obvious that the capacity attenuation rate per cycle of A900 is significantly lower and has exceedingly good cycle durability. Table 4 shows the comparison of the electrochemical performance of our work and other reported carbon materials used for LIBs. It is clear that our semicoke-based A900 is one of the most competitive alternatives for LIB anodes due to its delightful specific capacity and excellent cycle stability even under heavy current load, indicating good application prospects.

**Figure 6:**
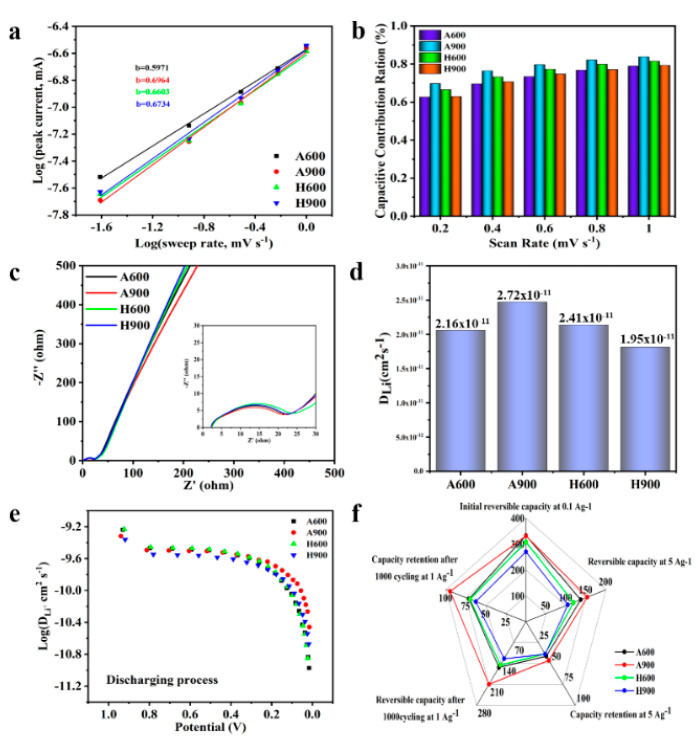
Log(i)–log(v) plots (**a**), capacitive contribution ratio of the samples at various scan rates (**b**), EIS of the samples after performing cyclic voltammetry (**c**), comparison of D_Li_ of the samples (**d**), the relation between D‘_Li_ and potential of the anodes during the discharging process at 0.1 A g^−1^ (**e**), and the comparison radar chart (**f**).

To further probe the kinetics of these prepared samples, the CV curves ranging from 0.2–1.0 mV s^−1^ were tested. As expressed in Figure S6, the shape of the CV curves at different sweep rates can be well preserved with the increasing scan rates. The relationship between peak current (i) and scan rate (v) can be hinted by Equation (6):

i = av^b^ (6)

where a and b are adjustable parameters [55]. The values of a and b are related to the intercept and slope, respectively, of the log(i)–log(v) profile. When b is close to 0.5, the capacity mainly comes from the diffusion-controlled intercalation process (DIP), whereas the surface-induced capacitive process (SCP) is dominant when b approaches 1 [13,14]. The calculated b values at 0.2 mV s^−1^ presented in Figure 6a are in the range of 0.59–0.70, suggesting that the coexistence of SCP and DIP in Li^+^ storage. A900 has the largest b value compared to other electrodes, indicating a more significant SCP in A900. To further quantify the SCP behavior, the following equation was applied [47].

i(ν) = k_1_ν + k_2_ν^1/2^ (7)

where k_1_ν and k_2_ν^1/2^ value stand for the capacitive contribution and diffusion-controlled contribution, respectively. As presented in Figure 6b, the SCP contributions of A600, A900, H600, and H900 increase with the increase in scan rate and reach up to 78.9%, 83.7%, 81.5%, and 79.3% at 1 mV s^−1^, respectively (Figure S7). It is observed that the capacitance contribution is dominant for the samples, confirming the fact that large surface area favors SCP contribution. Additionally, A900 delivers the highest SCP contribution at each scan rate, this phenomenon further endorses anodes with moderately ultramicropore structure, rich defects, and large interlayer spacing as being more conducive to Li^+^ adsorption and intercalation, enabling an improved capacity and rate capability.

EIS measurements were also conducted to further evaluate kinetics. As revealed in Figure 6c, all Nyquist plots are composed of the semicircle in the high-frequency range and the oblique line in the low-frequency region, which are in connection with the charge transfer resistance and the ion-diffusion process, respectively [13,56,57]. An electric equivalent circuit shown in Figure S8 was used to simulate the electrochemical process, and the fitting parameter values are presented in Table S7. R_s_, R_f_, and R_ct_ in the circuit correspond to the ohmic resistance of the electrolyte and the contact resistance in the cell, the resistance of the SEI layer, and the charge transfer resistance, respectively [41]. As shown in Table S7, the R_s_ values of all anodes are close, while those of R_f_ and R_ct_ show notable dissimilarity, indicating that R_s_ is not the key factor affecting battery performance for our samples in this work. By comparison, the A900 shows the lowest R_f_ (21.19 Ω) and R_ct_ (36.20 Ω). Smaller R_f_ and R_ct_ are feasible for faster electrochemical reaction and ion transport. Additionally, the Li^+^ diffusion coefficient (D_Li_) was further obtained based on the low-frequency region data. As shown in Figure 6d, A900 shows the highest D_Li_ of 2.72 × 10^−12^ cm^2^ s^−1^, suggesting superior ion diffusion behavior.

To further elucidate the transport kinetics, galvanostatic intermittent titration technique (GITT) was conducted by applying a pulse for 10 min and relaxing for 1 h at 0.1 A g^−1^. The Li^+^ diffusion coefficient (D‘_Li_) during the whole charge/discharge process can be found in Figure 6e. The calculated D‘_Li_ is in accordance with the results from the Nyquist plot analysis. Different potential range associates to a particular reaction mechanism with various diffusion coefficients [58]. Obviously, all samples have a relatively stable D’_Li_ voltage values in the range of 0.2–1.0 V, and the D‘_Li_ values decrease significantly in the voltage range of 0.01–0.20 V. This is due to the intercalation inside the graphite nanodomains being more sluggish than the adsorption processes on the defect sites. Furthermore, the diffusion coefficient D‘_Li_ of A900 is higher than that of the other three samples for voltage values of 0.01–0.20 V, further confirming the fact that the larger interlayer distance is conducive to the intercalation of Li^+^. The D’_Li_ value of H900 is lower, in the range of 0.2–1.0 V, which may be due to the obvious reduction of its surface oxygen functional groups and carbon defects. A radar chart is an ideal method for comparing multiple variables. As displayed in Figure 6f, although A600 shows slightly higher specific capacity, A900 displays the largest enclosed area compared with other samples, indicating the best overall performance.

**Figure 7:**
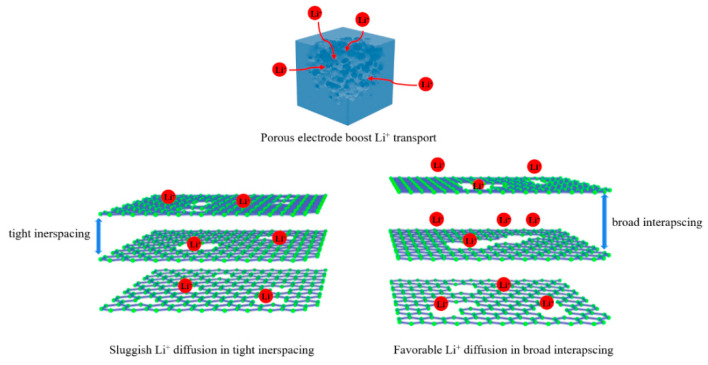
Illustration of Li^+^ storage behavior of semicoke-based carbon materials with different microstructures.

The model of Li^+^ storage for semicoke-based carbon materials is illustrated in Figure 7. The abundant mesopores provide affluent channels for rapid transport of Li^+^ ions, resulting in plentiful shortened ion pathways. The ultramicropore (<1 nm) and carbon defects (sp^3^ hybridized carbon) provide extra adsorption sites for Li^+^, giving rise to the increase in specific capacity. The enlarged interlayer distance reduces the diffusion resistance of Li^+^ between graphite layers, leading to accelerated ion kinetics. On account of the merits mentioned above, the semicoke-derived porous carbon anode shows outstanding electrochemical performance in Li^+^ storage.

## 4. Conclusions

In summary, interlayer-modulated SC-based porous carbons were prepared via facile pyrolytic tactics under various atmospheres. Temperature and atmosphere have a non-negligible impact on the regulation of the structure (especially for the modulation of turbostatic stacking structure) and the electrochemical performance of SC-derived carbons. Thanks to the high accessible specific surface and developed porous structure, the electrodes exhibit boosted Li^+^ adsorption capacity and ion transportation pathways, while the enlarged interlayer distance facilitates diffusion rate between carbon layers. Thus, SCP and DIP behaviors work together to improve Li^+^ storage performance. Furthermore, the increased Li ion diffusion coefficient calculated from GITT and EIS further verifies the increased ion kinetics. The combination of designed interlayer distance, optimized pore structure, and short-ordered defective domains endows A900 with superior overall Li^+^ storage behavior. Concretely, A900 delivers a high specific capacity of 331.1 mAh g^−1^ at the current density of 0.1 A g^−1^ coupled with a good specific capacity retention (153.2 mAh g^−1^ at 5 A g^−1^). Additionally, it also shows excellent cycling stability (94.8% capacity retention even after 1000 consecutive cycles at 1 A g^−1^) with an ultra-low capacity fading rate of 0.005% per cycle. Our research has proved that SC has outstanding potential as an anode precursor for LIBs.

## Figures and Tables

**Table 1:** The structural parameters calculated based on the XRD patterns.

Sample	2θ_002_ (°)	d_002_ (nm)	FWHM _(002)_	2θ_100_ (°)	FWHM _(100)_	L_a_ (nm)	L_c_ (nm)	I_g_	R
A600	24.59	0.3616	5.88	43.22	4.27	4.0896	1.3823	60.47	1.7447
A900	24.46	0.3641	5.56	43.37	3.86	4.5263	1.4614	55.09	1.8182
H600	24.60	0.3612	5.79	43.12	4.09	4.2681	1.4039	64.37	1.7636
H900	24.54	0.3615	5.48	43.28	3.42	5.1071	1.4832	68.60	1.8458

**Table 2:** The calculated results of F_1_, F_2_, F_3_, and F_4_.

Sample	F_1_	F_2_	F_3_	F_4_
A600	0.1923	2.3682	2.6609	3.4815
A900	0.1801	2.5725	2.9001	3.6318
H600	0.1961	2.2871	2.5880	3.3320
H900	0.1899	2.4966	2.7709	3.4929

**Table 3:** The relative content of the C 1s peaks in deconvoluted A600, A900, H600, and H900.

Assignment	C1	C2	C3	C4	C5
	sp^2^ C	sp^3^ C	C-O	C=O	π-π*
A600	54.26	21.80	10.47	8.94	4.53
A900	51.23	25.14	10.04	7.63	5.95
H600	59.18	18.60	9.99	7.72	4.51
H900	54.93	22.42	9.57	7.56	5.53

**Table 4:** Electrochemical performance comparison between A900 and other anodes from previous literature.

Materials	Capacity (mAh g^−1^)	Rate Capacity (mAh g^−1^)	Capacity Retention	Ref.
A900	331 (0.1 A g^−1^)	153 (5 A g^−1^)	94.8% (1000 cycles, 1 A g^−1^)	This work
BCG-2800	310 (0.0372 A g^−1^)	135 (1.86 A g^−1^)	95.3% (100 cycles, 0.744 A g^−1^)	[52]
Porous carbon	338 (0.05 A g^−1^)	81 (5 A g^−1^)	50.4% (800 cycles, 5 A g^−1^)	[37]
LSHC-P200	411 (0.1 A g^−1^)	32 (5 A g^−1^)	62.5% (400 cycles, 0.5 A g^−1^)	[40]
CG-2500	282 (0.1 A g^−1^)	43 (5 A g^−1^)	100% (1000 cycles, 0.5 A g^−1^)	[53]
BCNF	321 (0.0372 A g^−1^)	50 (0.744 A g^−1^)	77% (50 cycles, 0.0372 A g^−1^)	[54]

## Data Availability

The data are available upon reasonable request from the corresponding author.

## Supplementary Materials

The following supporting information can be downloaded at: https://www.mdpi.com/article/10.3390/nano12224067/s1, Figure S1: EDS elemental mapping results of semi-cokes before (a,c,d) and after purification (b,d,f); Figure S2: SEM image (a) and TEM image (b) of SC; Figure S3: The calculated R value for A600 (a), A900 (b), H600 (c) and H900 (d); Figure S4: The CV curves of A600 (a), A900 (b), H600 (c) and H900 (d) at 0.2 mV s^−1^; Figure S5: Charge/discharge profiles of A600 (a), A900 (b), H600 (c) and H900 (d) at 0.1 A g^−1^; Figure S6: CV curves of A600 (a), A900 (b), H600 (c) and H900 (d) at different scan rates; Figure S7: Separation of storage contribution from the capacitance and diffusion-controlled process at 1 mV s^−1^ (the former is marked with red areas) of A600 (a), A900 (b), H600 (c) and H900 (d); Figure S8: Relevant equivalent circuit; Table S1: Proximate analysis of semi-coke bulks (wt.%); Table S2: Element content of EDS analysis for semi-coke; Table S3: Elemental analysis of SC, A600, A900, H600 and H900 (wt.%); Table S4: Summary of textural parameters obtained from nitrogen adsorption analysis; Table S5: The percent content of C and O from XPS overall scans (at.%); Table S6: The values in coulombic efficiency for first ten cycles; Table S7: Fitting results of Nyquist plots.
