# Supplementary material for: Coal-Based Semicoke-Derived Carbon Anode Materials with Tunable Microcrystalline Structure for Fast Lithium-Ion Storage
Source: Nanomaterials (Basel). 2022 Nov 18;12(22):4067. doi: 10.3390/nano12224067 (PMC9699443; doi:10.3390/nano12224067)
Supplement: Supplementary file 1 [file nanomaterials-12-04067-s001.zip › nanomaterials-2001285-supplementary.pdf]

nanomaterials

Supplementary Materials

Coal-Based Semicoke-Derived Carbon Anode Materials with Tunable Microcrystalline Structure for Fast Lithium-Ion Storage

Yaxiong Liu [1,2], Xing Guo [1,2], Xiaodong Tian [1,*] and Zhanjun Liu [1,2,*]

[1] CAS Key Laboratory of Carbon Materials, Institute of Coal Chemistry, Chinese Academy of Sciences, Taiyuan 030001, China
[2] Center of Materials Science and Optoelectronics Engineering, University of Chinese Academy of Sciences, Beijing 100049, China
* Correspondence: tianxiaodong@sxicc.ac.cn (X.T.); zjliu03@sxicc.ac.cn (Z.L.)

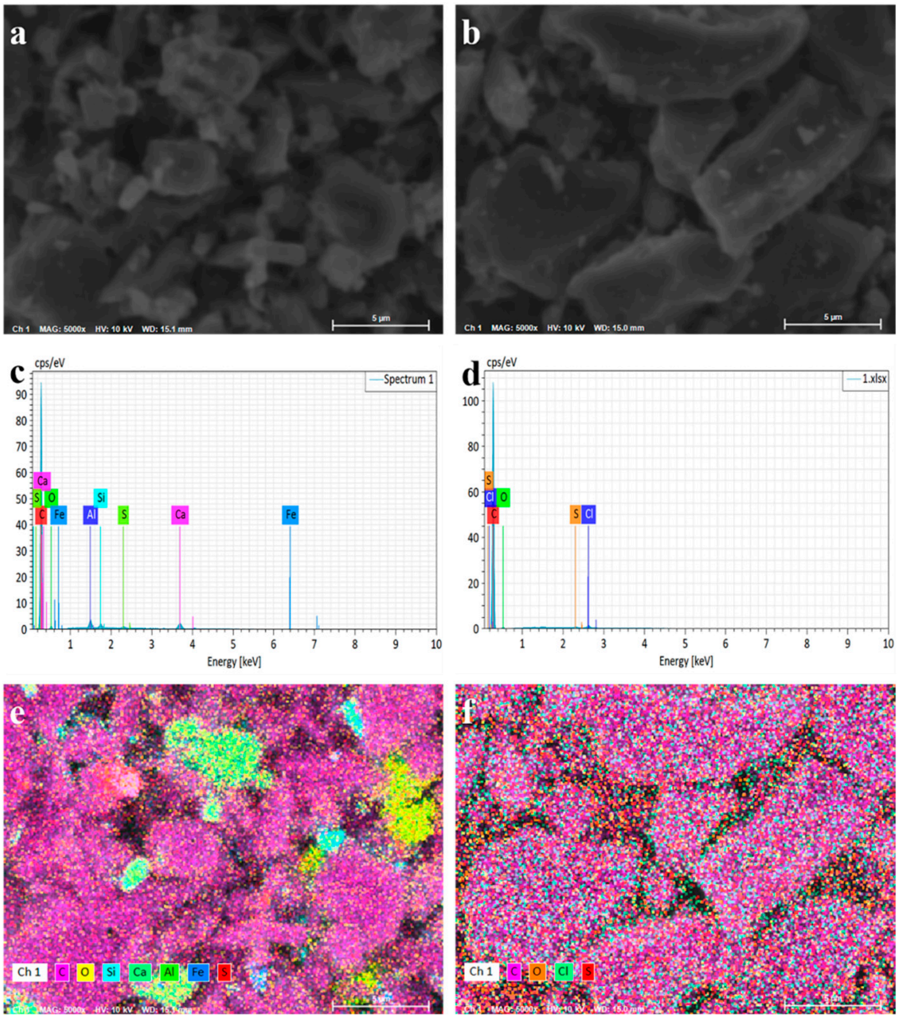

Figure S1. EDS elemental mapping results of semi-cokes before (**a**,**c**,**d**) and after purification (**b**,**d**,**f**).

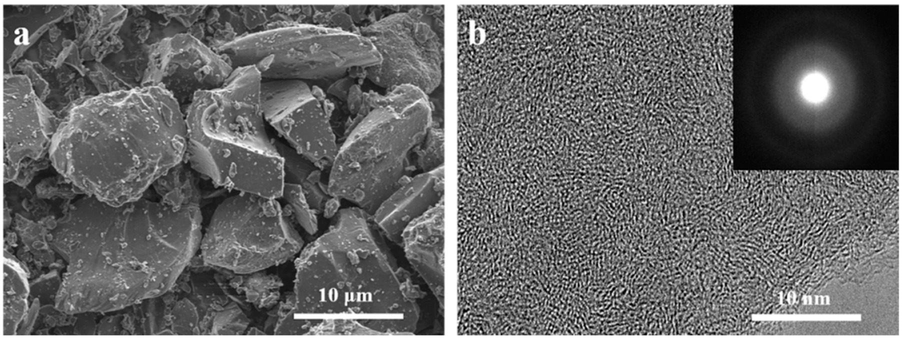

Figure S2. SEM image (**a**) and TEM image (**b**) of SC.

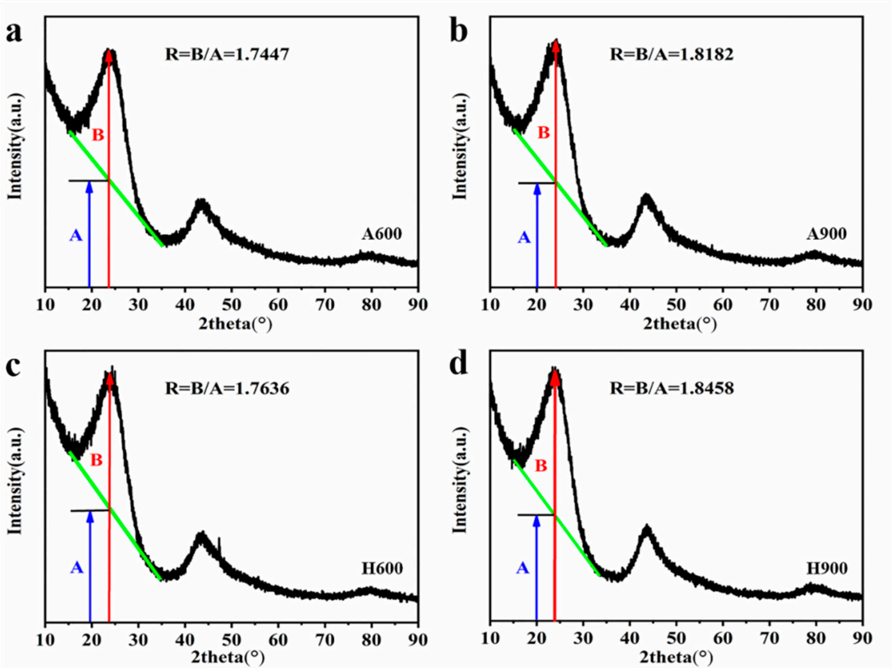

Figure S3. The calculated R value for A600 (**a**), A900 (**b**), H600 (**c**) and H900 (**d**).

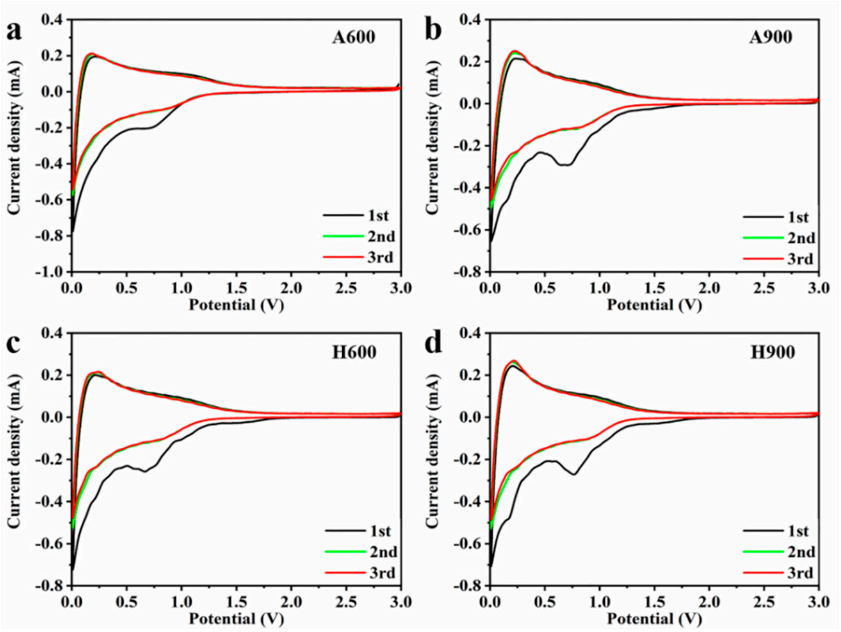

Figure S4. The CV curves of A600 (**a**), A900 (**b**), H600 (**c**) and H900 (**d**) at 0.2 mV s^{-1}.

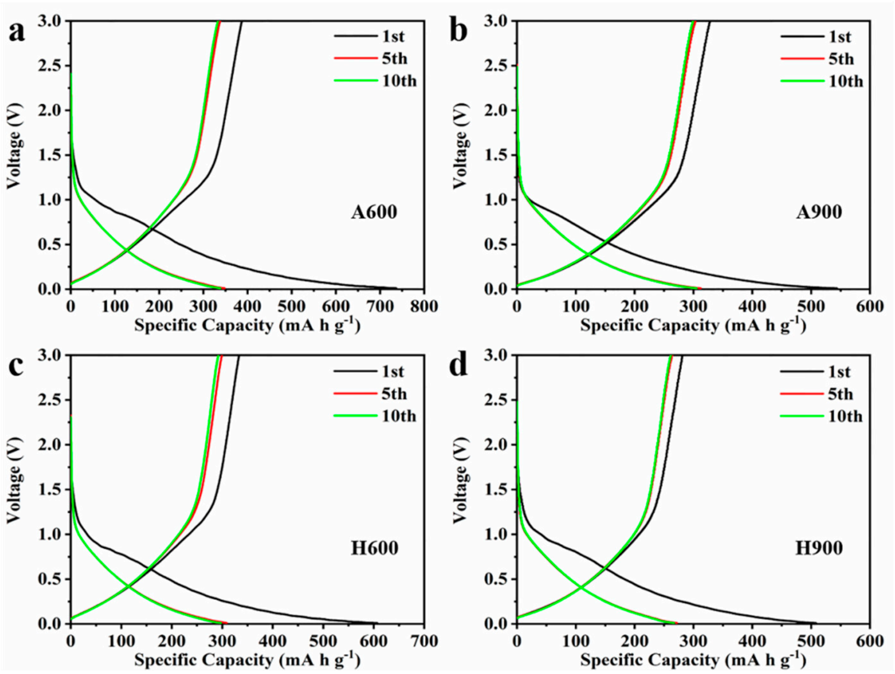

Figure S5. Charge/discharge profiles of A600 (**a**), A900 (**b**), H600 (**c**) and H900 (**d**) at 0.1 A g^{-1}.

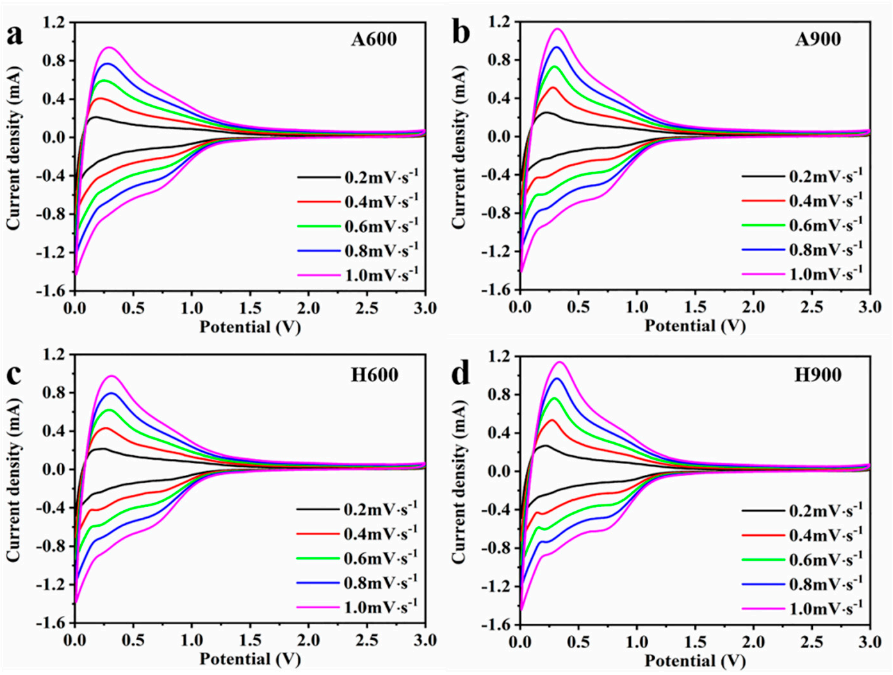

Figure S6. CV curves of A600 (**a**), A900 (**b**), H600 (**c**) and H900 (**d**) at different scan rates.

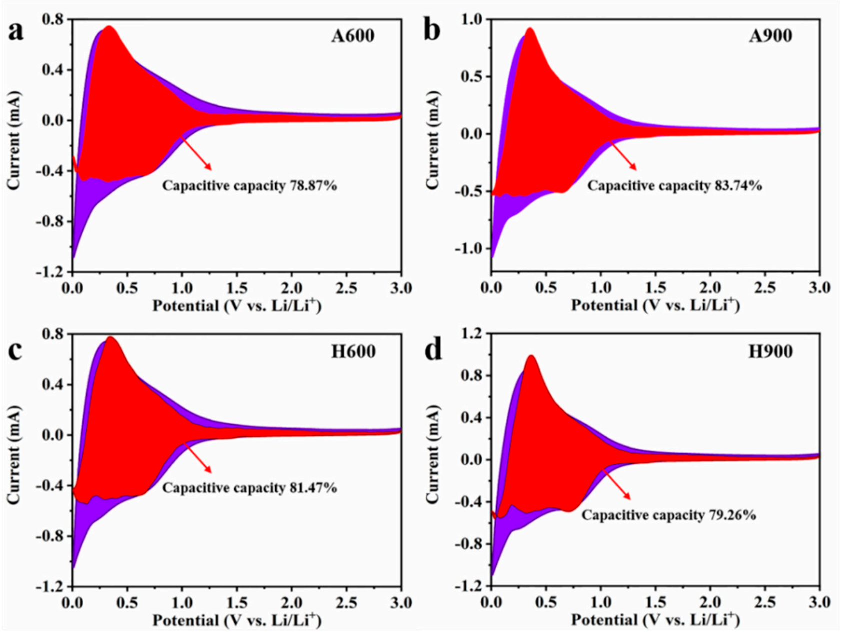

Figure S7. Separation of storage contribution from the capacitance and diffusion-controlled process at 1 mV s^{-1} (the former is marked with red areas) of A600 (**a**), A900 (**b**), H600 (**c**) and H900 (**d**).

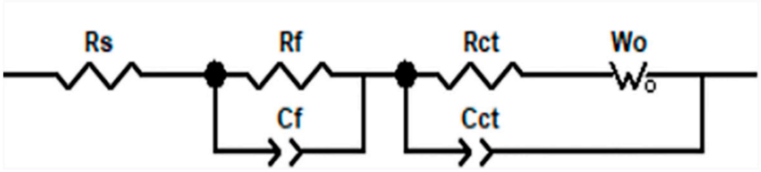

Figure S8. Relevant equivalent circuit.

Table S1. Proximate analysis of semi-coke bulks (wt.%).

Proximate analysis				
M_{ad}	A_d	V_d	S_d	FC_d
5.78	4.80	4.66	0.23	84.53

Note: M: moisture, A: ash, V: volatile, S: sulfur, FC: fixed carbon, ad: Air-dried basis, d: dry basis.

Table S2. Element content of EDS analysis for semi-coke.

Element	Semi-coke		Purified semi-coke	
	Mass (%)	At. (%)	Mass (%)	At. (%)
C	79.67	86.57	91.56	93.97
O	13.45	10.97	7.30	5.63
Ca	3.64	1.18	-	-
Al	1.18	0.57	-	-
Si	0.77	0.36	-	-
Fe	1.01	0.24	-	-
S	0.28	0.11	0.26	0.1
Cl	-	-	0.87	0.3

Table S3. Elemental analysis of SC, A600, A900, H600 and H900 (wt.%).

Sample	C	H	S	O
SC	89.86	1.26	0.08	8.80
A600	93.34	1.28	0.23	5.15
A900	95.09	0.86	0.28	3.77
H600	94.73	1.23	0.27	3.77
H900	96.02	0.78	0.32	2.88

Table S4. Summary of textural parameters obtained from nitrogen adsorption analysis.

Sample	BET (m^2 g^{-1})	Pore volume (cm^3 g^{-1})	Pore size (nm)
A600	195.4	0.108	2.215
A900	226.3	0.128	2.255
H600	216.7	0.123	2.262
H900	184.9	0.110	2.386

Table S5. The percent content of C and O from XPS overall scans (at.%).

Element	A600	A900	H600	H900
C	96.47	98.23	97.11	98.66
O	3.53	1.77	2.89	1.34

Table S6. The values in coulombic efficiency for first ten cycles.

Sample	1st cycle	2nd cycle	3rd cycle	5th cycle	10th cycle
A600	52.54	92.78	95.59	96.98	98.41
A900	55.66	93.38	95.98	97.64	98.45
H600	54.94	93.86	96.40	96.89	98.26
H900	55.36	93.13	95.88	97.06	98.10

Table S7. Fitting results of Nyquist plots.

Sample	Rs	Rf	Rct
A600	1.90	21.87	47.41
A900	2.02	21.19	36.20
H600	2.20	23.20	36.70
H900	1.91	22.25	48.42
